# Who will repair the next open inguinal hernia? Educational consequences of declining open surgery volumes

**DOI:** 10.1007/s10029-026-03801-9

**Published:** 2026-07-27

**Authors:** Jacob Rosenberg, Kristoffer Andresen

**Affiliations:** Department of Surgery, Center for Perioperative Optimization, Herlev Hospital, University of Copenhagen, Herlev, DK-2730 Denmark

**Keywords:** Inguinal hernia, Lichtenstein repair, Laparoscopic hernia repair, Surgical training, Centralisation, Denmark

## Abstract

**Purpose:**

Over the past two decades, laparoscopic repair has increasingly replaced open techniques for elective groin hernia surgery in Denmark. This transition has profoundly altered the case mix and surgical exposure. The implications for surgical training, competence maintenance, and future service provision remain largely unexplored.

**Methods:**

Using prospectively collected data from the Danish Inguinal Hernia Database, we conducted an updated nationwide cohort analysis of elective groin hernia repairs from 1998 to 2025. Surgical techniques were classified as open (primarily Lichtenstein repair) or minimally invasive (primarily laparoscopic TAPP repairs, including robot-assisted). Annual procedure volumes, departmental distribution in 2025, and cumulative reoperation rates after Lichtenstein and TAPP repairs were analyzed.

**Results:**

A total of 296,864 unique elective repairs in 277,666 groins were analyzed. Open repair accounted for only 19% of unilateral repairs in 2025, while 80% were performed minimally invasive. Open procedures were unevenly distributed across departments: of 40 Danish departments performing Lichtenstein repair in 2025, only four performed more than 100 repairs annually, while 18 performed fewer than 20. Cumulative reoperation rates after Lichtenstein repair have increased significantly across consecutive five-year periods, whereas reoperation rates after TAPP repair did not show a comparable increase.

**Conclusion:**

Open inguinal hernia repair is now a low-volume operation, with declines in long-term outcomes. Maintaining broad competence in open repair through traditional surgical training pathways no longer seems realistic. Centralizing open hernia surgery at dedicated centers or with specialized surgeons, along with a stronger emphasis on laparoscopic training, may be necessary to preserve quality, safety, and training opportunities.

## Introduction

Groin hernia repair is among the most commonly performed general surgical procedures worldwide and has long played a central role in surgical education [1]. For decades, open anterior mesh repair – most notably the Lichtenstein technique – has been considered a foundational operation for surgical trainees, providing structured exposure to anatomy, tissue handling, and perioperative decision-making [2]. International guidelines continue to recommend open mesh repair as one of two acceptable standards for primary groin hernia, alongside laparoscopic techniques [3]. Over the past two decades, laparoscopic techniques have increasingly replaced open repair for unilateral and bilateral inguinal hernias. This shift has been driven by accumulating evidence showing reduced postoperative pain, faster recovery, and a lower risk of chronic pain after laparoscopic repair, particularly for unilateral primary hernias [4], as well as guideline recommendations favoring minimally invasive approaches in defined patient groups [3]. In countries with mature hernia registries and structured quality initiatives, including Denmark, the transition has occurred rapidly and on a national scale [5, 6]. Nationwide analyses have shown a steady decline in the use of open repair, with laparoscopic techniques now accounting for the majority of elective groin hernia operations [7, 8].

While the clinical consequences of this development have been extensively studied, its educational implications have received limited attention. Surgical competence is inherently volume-dependent, and even technically standardized operations require repeated exposure for skill acquisition and retention. Recent Danish data show that surgeon experience – measured by cumulative case volume – is independently associated with intraoperative quality markers, such as nerve identification during Lichtenstein repair [9]. As the absolute number of open groin hernia repairs declines, opportunities for trainees to acquire and maintain proficiency in open techniques are diminishing. At the same time, the residual open procedures are no longer representative of routine cases. They increasingly involve older, comorbid patients with prior abdominal surgery, recurrent disease, or complex anatomy, and may require open preperitoneal or other alternative open mesh approaches that are rarely performed [10]. Such cases are inherently less suitable as trainee operations and carry a higher risk profile, further challenging the assumption that open hernia repair can continue to serve as a basic educational procedure.

This raises a fundamental question for contemporary hernia surgery: who should perform open inguinal hernia repairs in the future, and how should surgical education be organized in an era when open repair is no longer routine? If open groin hernia surgery becomes a low-volume procedure performed by a few surgeons, traditional decentralized training models may no longer be appropriate. Instead, alternative organizational and educational strategies may be required to ensure patient safety, procedural quality, and meaningful surgical training [11].

The aim of this study was to provide an updated nationwide analysis of surgical techniques for elective groin hernia repair in Denmark, with a particular focus on current absolute and departmental volumes of open repairs and on long-term reoperation rates. By placing these findings in an educational context, we explore their implications for surgical training and inform future discussions on how open groin hernia repair should be organized and taught.

## Methods

### Study design and data source

This nationwide cohort study used prospectively collected data from the Danish Inguinal Hernia Database for the period from 1 January 1998 through 31 December 2025 [12]. The database has mandatory national coverage and includes all elective groin hernia repairs performed in both public and private hospitals in Denmark. Data are entered by the operating surgeon at the time of surgery and are supplemented by automated linkage to the Danish National Patient Registry, which ensures high completeness and validity [13]. The Danish digital infrastructure supporting register-based research has been described in detail elsewhere [14].

### Study population

We included all adult patients (≥ 18 years) undergoing elective unilateral or bilateral groin hernia repair during the study period. Procedures registered as emergencies and records lacking essential operative information (in particular, missing side data or duplicate registrations) were excluded. After exclusions, 296,864 unique elective repairs in 277,666 groins were available for analysis. For patients undergoing bilateral repair on the same date, both groins were treated as a single index procedure; sequential repairs of the contralateral groin on different dates were registered as separate entries.

### Classification of surgical technique

Surgical techniques were categorized by the primary operative approach and grouped into two main categories in accordance with international and Danish consensus definitions [11, 15]:

1. Open repair, including Lichtenstein repair, modified Lichtenstein, Onstep, open preperitoneal repair, other open mesh-based techniques, and open non-mesh repair.
2. Minimally invasive (laparoscopic) repair, including transabdominal preperitoneal (TAPP) and totally extraperitoneal (TEP) repairs, and their corresponding robot-assisted variants.

Robot-assisted laparoscopic procedures were grouped with minimally invasive techniques and also reported descriptively.

### Outcomes

The primary outcome was the annual number and proportion of open versus minimally invasive groin hernia repairs over time. Secondary outcomes were the absolute nationwide number of open repairs per year, the distribution of open repairs across surgical departments in 2025, and cumulative reoperation rates for recurrence after primary unilateral Lichtenstein and TAPP repair, stratified by five-year periods. Reoperation for recurrence was defined as a subsequent groin hernia repair in the same groin irrespective of the hernia anatomy (direct, indirect, femoral). For bilateral repairs a reoperation for recurrence in either groin was considered an event. Reoperation for ipsilateral recurrence was used as a validated surrogate for recurrence, as established in Danish and Swedish registry research [6, 16].

### Statistical analysis

Descriptive statistics summarized patient characteristics and operative techniques. Continuous variables are presented as means with standard deviations, and categorical variables as counts with percentages. Trends over time are illustrated using absolute numbers and proportions, presented separately for unilateral and bilateral repairs where relevant. Time-to-event analyses for reoperation were performed using the Kaplan–Meier method, with 95% confidence intervals shown as shaded areas; differences between five-year strata were assessed using the log-rank test, with *P* < 0.05 considered statistically significant. Analyses were performed in R (R Core Team, R Foundation for Statistical Computing, Vienna, Austria) using RStudio version 2024.04.2 and the packages dplyr, stringr, tidyverse, haven, ggplot2, survival, and survminer.

### Ethical considerations

Under Danish law, register-based studies using anonymized data do not require approval from a regional ethics committee or informed patient consent. The study was approved by the Danish Data Protection Agency (record number p-2026-20752) and by the Clinical Quality Development Program (Regionernes Kliniske Kvalitetsudviklingsprogram, RKKP) in the Regions of Denmark.

## Results

### Study population

**Fig. 1:**
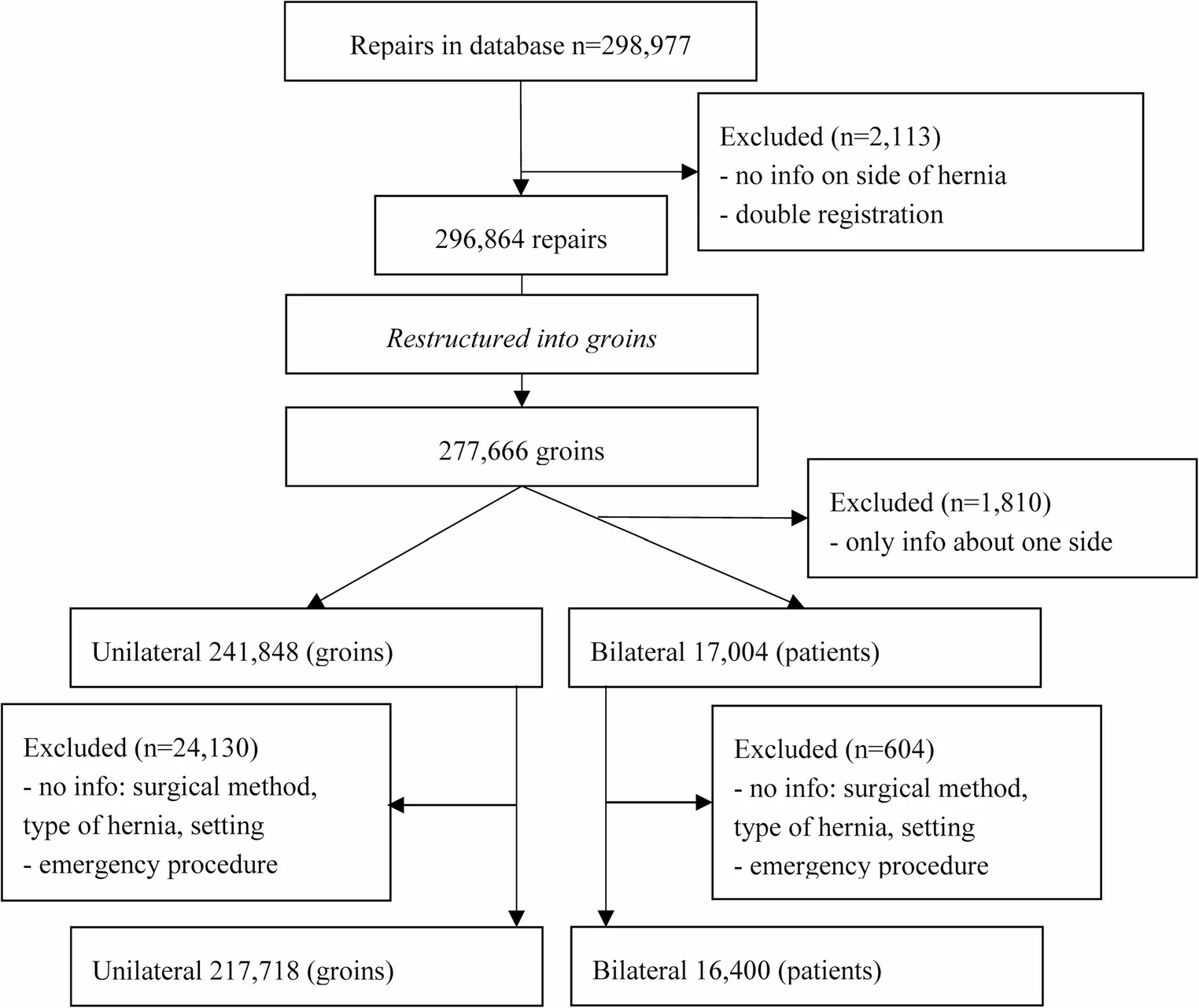
Flowchart of patient inclusion and exclusion in the Danish inguinal hernia database, 1998–2025. Of 298,977 registered repairs, 2,113 were excluded for missing information on side or duplicate registration, leaving 296,864 unique elective repairs in 277,666 groins for analysis

A nationwide cohort of 296,864 unique elective groin hernia repairs in 277,666 groins was identified from the Danish Inguinal Hernia Database for the period 1998–2025. The flow of records through identification and exclusion is shown in Fig. 1. Demographic characteristics for the full study period, the most recent five-year period (2021–2025), and for the year 2025 are presented in Table 1. The cohort was predominantly male (88–91%), with a mean age of 59–63 years across periods, and reflected the national distribution of elective groin hernia surgery in Denmark. No substantial change in baseline patient characteristics was observed over time.

**Table 1:** Demographics and operative methods for elective groin hernia repairs registered in the Danish inguinal hernia database, stratified by laterality, for the full study period (1998–2025), the earliest five-year period (1998–2002), the most recent five-year period (2021–2025), and for 2025. The variable “no hernia” could only be registered until 2012

Demographics	1998–2025		1998–2002		2021–2025		2025	
	Unilateral	Bilateral	Unilateral	Bilateral	Unilateral	Bilateral	Unilateral	Bilateral
N	217,718	16,400	43,967	1,539	39,132	4,443	8,614	1,049
Age (years), mean [SD]	59 [16]	60 [14]	57 [16]*	59 [14]*	62 [15]*	62 [14]*	63 [15]	62 [14]
Males, n [%]	198,039 [91]	14,928 [91]	40,642 [92]*	1,467 [95]*	34,772 [89]*	3,862 [87]*	7,581 [88]	894 [85]
General anaesthesia, n [%]	172,321 [79]	15,986 [97]	27,102 [62]*	1,244 [81]*	37,309 [95]*	4,427 [100]*	8,261 [96]	1,048 [100]
Type of hernia, n [%]								
Combined	1,613 [1]	304 [2]	127 [0]	5 [0]	631 [2]	112 [3]	168 [2]	39 [4]
Femoral	4,693 [2]	339 [2]	688 [2]	14 [1]	1,068 [3]	124 [3]	264 [3]	31 [3]
Inguinal	199,303 [92]	14,622 [89]	40,678 [93]*	1,417 [92]*	35,429 [91]*	3,916 [88]*	7,953 [92]	916 [87]
No hernia	690 [0]	32 [0]	309 [1]	13 [1]	0 [0]	0 [0]	0 [0]	0 [0]
Pantaloon	11,419 [5]	1,103 [7]	2,165 [5]	90 [6]	2,004 [5]	291 [7]	229 [3]	63 [6]
Method, n [%]								
Combined infra- and supraligament	723 [0]	2 [0]	306 [1]	2 [0]	6 [0]	0 [0]	0 [0]	0 [0]
Laparoscopic, converted	171 [0]	8 [0]	0 [0]	0 [0]	132 [0]	5 [0]	26 [0]	1 [0]
Lichtenstein	129,977 [60]	1,122 [7]	28,838 [66]*	559 [36]*	9,625 [25]*	31 [1]*	1,652 [19]	2 [0]
Modified Lichtenstein	15 [0]	0 [0]	0 [0]	0 [0]	15 [0]	0 [0]	2 [0]	0 [0]
Onstep	1,182 [1]	6 [0]	0 [0]	0 [0]	2 [0]	0 [0]	0 [0]	0 [0]
Open infraligament	1,095 [1]	4 [0]	351 [1]	2 [0]	7 [0]	0 [0]	0 [0]	0 [0]
Open mesh, unspecified	12,232 [6]	363 [2]	7,826 [18]	295 [19]	105 [0]	4 [0]	7 [0]	0 [0]
Open non-mesh	6,862 [3]	115 [1]	5,492 [12]	108 [7]	27 [0]	0 [0]	6 [0]	0 [0]
Robot, converted to laparoscopic	8 [0]	0 [0]	0 [0]	0 [0]	8 [0]	0 [0]	2 [0]	0 [0]
Robot, converted to open	5 [0]	0 [0]	0 [0]	0 [0]	5 [0]	0 [0]	3 [0]	0 [0]
TAPP	62,807 [29]	13,994 [85]	1,066 [2]*	535 [35]*	27,588 [70]*	3,971 [89]*	6,444 [75]	910 [87]
TAPP, robot-assisted	1,850 [1]	544 [3]	0 [0]	0 [0]	1,332 [3]	398 [9]	382 [4]	131 [12]
TAPP robot, converted	7 [0]	3 [0]	0 [0]	0 [0]	3 [0]	2 [0]	0 [0]	0 [0]
TEP	769 [0]	238 [1]	88 [0]	38 [2]	265 [1]	32 [1]	83 [1]	5 [0]
TEP, robot-assisted	15 [0]	1 [0]	0 [0]	0 [0]	12 [0]	0 [0]	7 [0]	0 [0]
Setting, n [%]								
Elective	217,718 [100]	16,400 [100]	43,967 [100]	1,539 [100]	39,132 [100]	4,443 [100]	8,614 [100]	1,049 [100]

* indicate p-values < 0.05 for comparisons between 1998–2002 and 2021–2025 for unilateral and bilateral repairs, respectively

### Trends in surgical technique

**Fig. 2:**
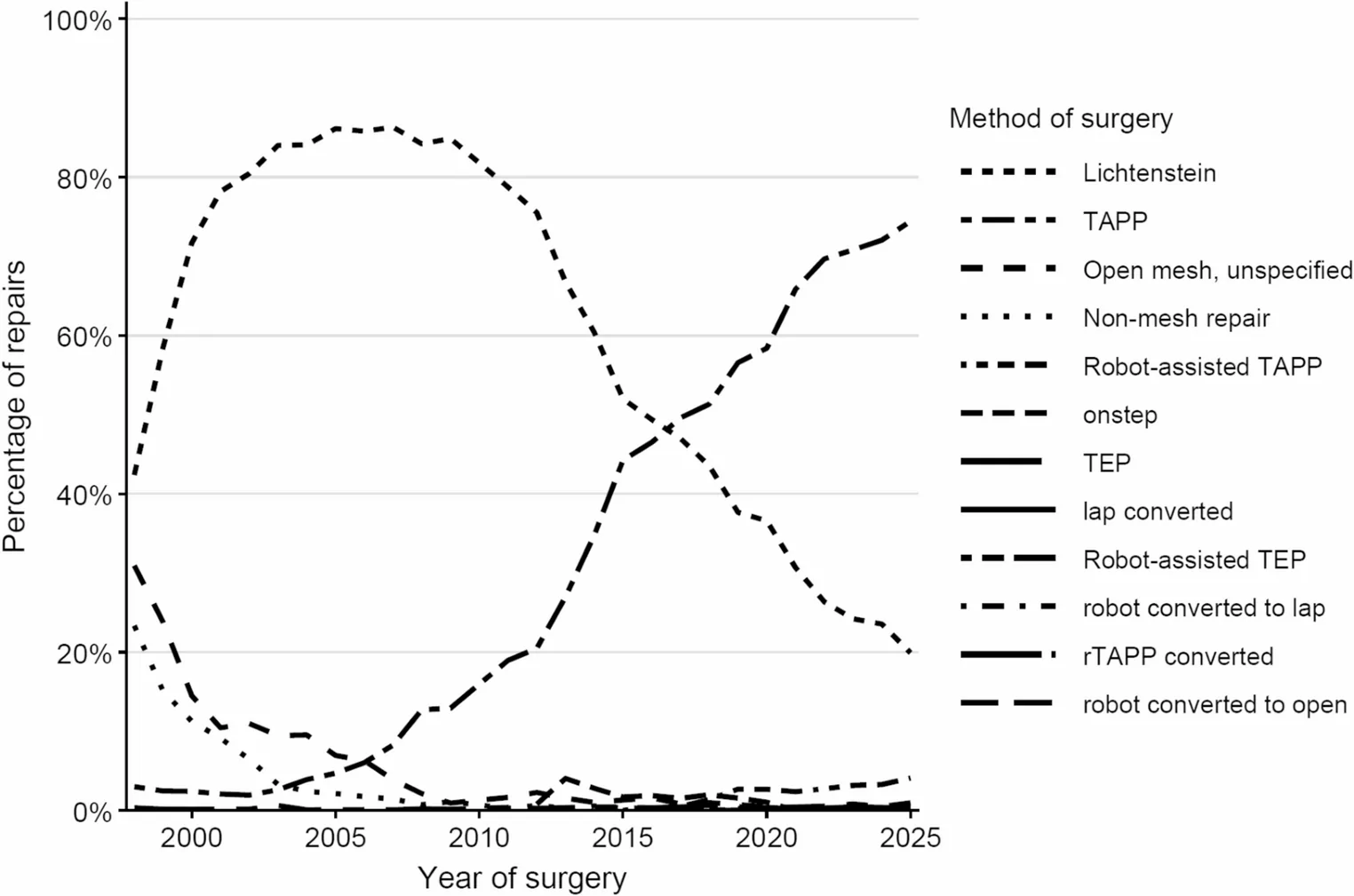
Surgical approach for primary unilateral inguinal hernias performed in elective settings in Denmark, 1998–2025

**Fig. 3:**
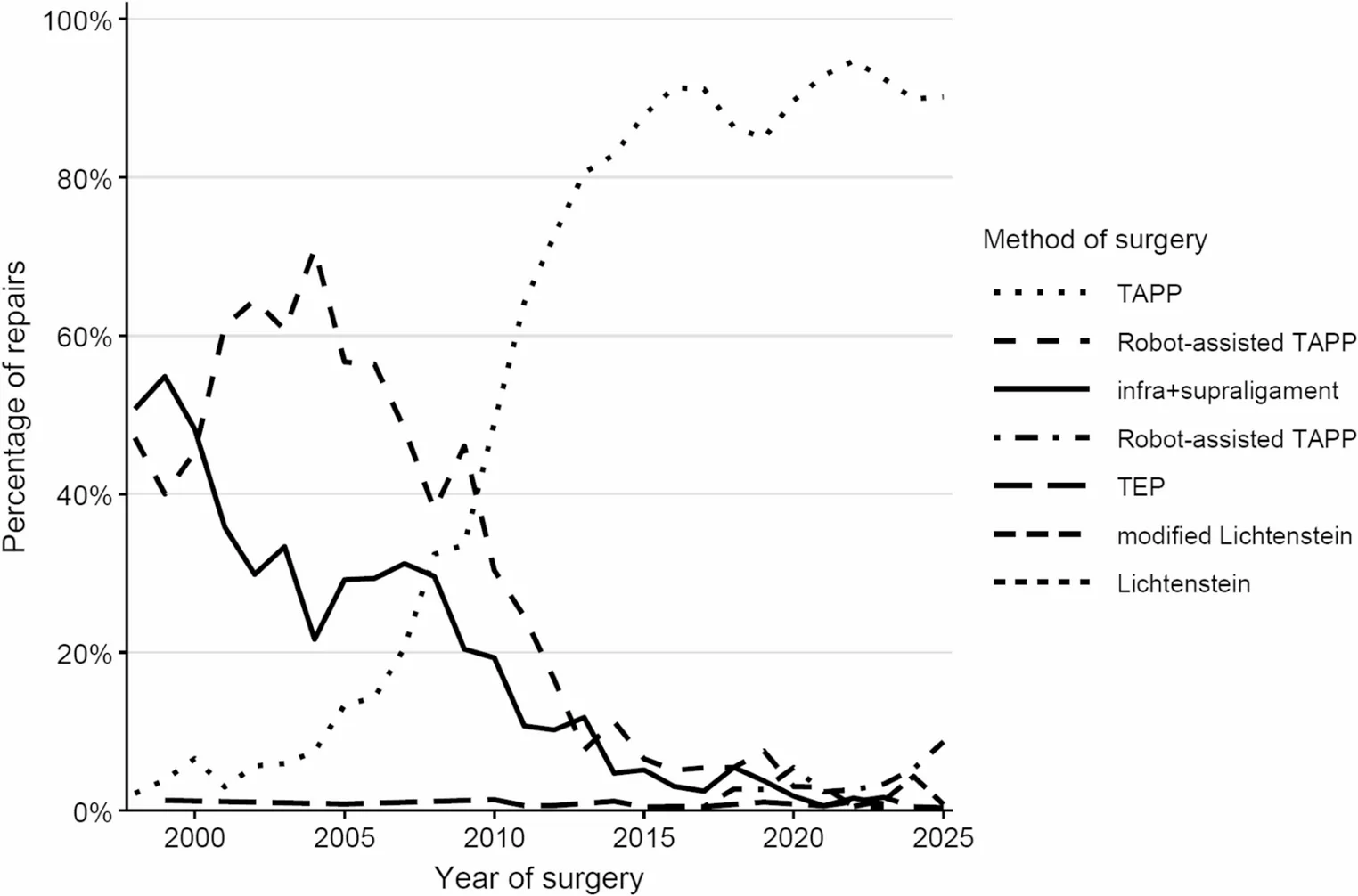
Surgical approach for primary unilateral femoral hernias performed in elective settings in Denmark, 1998–2025

A marked and sustained shift in operative technique was observed over the study period for both inguinal (Fig. 2) and femoral (Fig. 3) hernias. Laparoscopic repair has progressively replaced open repair and, in 2025, accounted for 80% of unilateral primary inguinal hernia repairs, while Lichtenstein repair accounted for only 19% (Table 1). For bilateral inguinal hernias, the transition was even more pronounced: in 2025, essentially all bilateral repairs were performed laparoscopically (predominantly TAPP), and open techniques had effectively been abandoned.

### Absolute volume of open repair

Despite a relatively stable overall national volume of elective groin hernia surgery, the absolute number of Lichtenstein repairs has continued to decline, from 1,948 procedures in 2021 to 1,654 in 2025. Other open mesh techniques (open mesh unspecified, Onstep, modified Lichtenstein, open preperitoneal) and open non-mesh repair together accounted for only a few procedures per year nationally (Table 1). The aggregated annual nationwide volume of open groin hernia repair is therefore now low in both relative and absolute terms. The non-Lichtenstein open techniques were performed in such negligible and diminishing numbers throughout the study period (Table 1) that they contribute little to either contemporary practice or to training capacity. The subsequent analyses of departmental distribution and long-term outcomes therefore focus on Lichtenstein repair as the representative open technique, while the remaining open methods are retained in Table 1 only for completeness and to document the full open volume.

### Distribution of open repair across departments

**Fig. 4:**
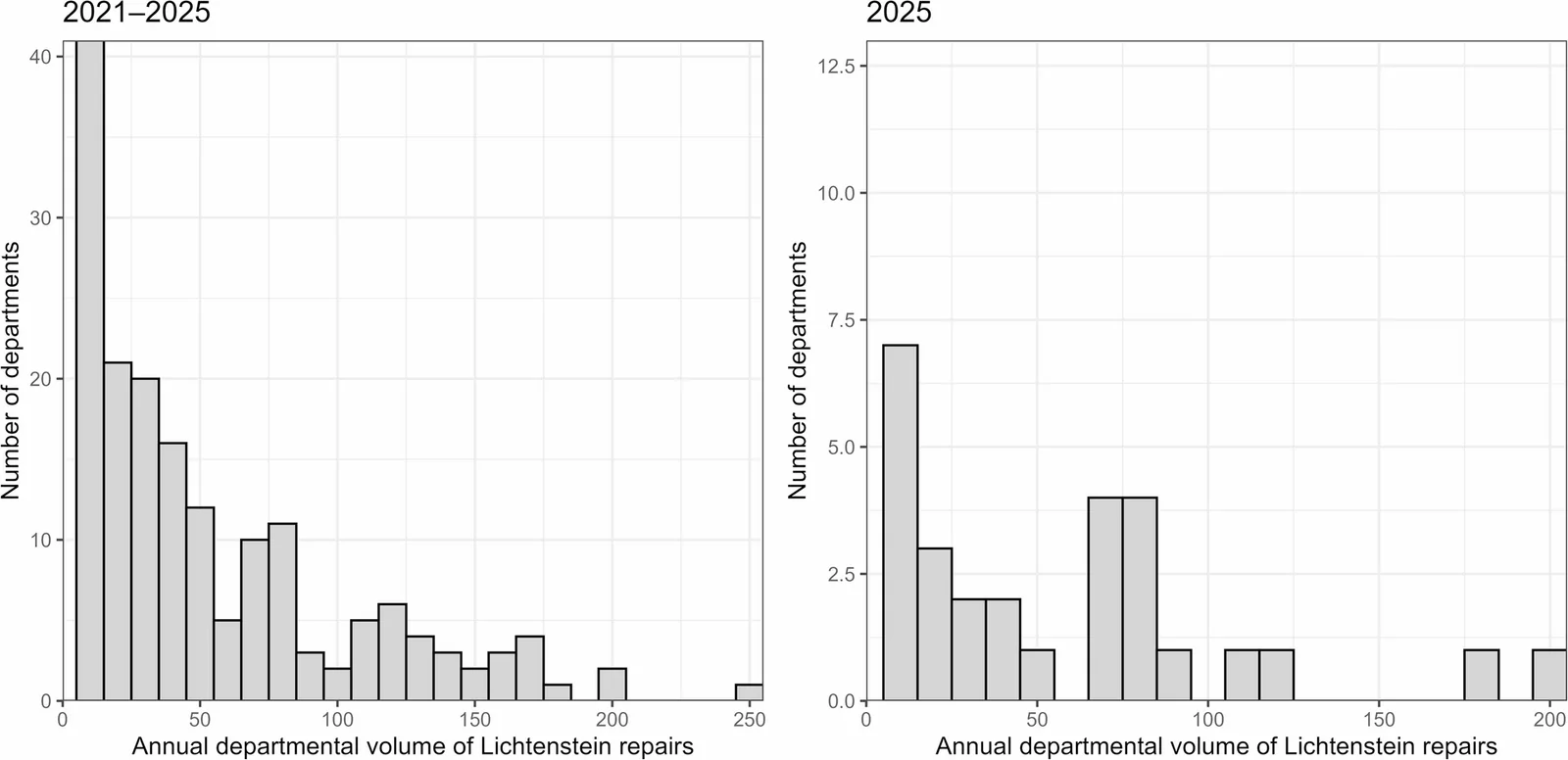
Histograms showing the distribution of annual departmental volume of elective Lichtenstein repair in Denmark, for the most recent five-year period (2021–2025) and for the index year 2025. Each bar represents the number of departments within a defined annual-volume band

Open groin hernia repair was unevenly distributed across surgical departments. In 2025, elective Lichtenstein repair was performed in 40 Danish departments. Only four departments performed more than 100 Lichtenstein repairs that year, while 18 departments performed fewer than 20 such repairs annually. As a result, the median departmental exposure to open groin hernia surgery is well below thresholds typically associated with sustained procedural competence, and exposure for individual surgeons – and for trainees rotating through these departments – is correspondingly limited. The full distribution of annual departmental Lichtenstein volume is shown as a histogram for both the most recent five-year period (2021–2025) and the index year (2025) in Fig. 4. It should be emphasised that the database does not record the number of surgeons performing open repair within each department. Departmental volume is therefore an imperfect proxy for individual surgeon exposure: a department performing 50–100 repairs annually with only one or two dedicated hernia surgeons may sustain reasonable individual caseloads, whereas the same volume shared across many rotating surgeons and trainees would not. The departmental thresholds used here should accordingly be interpreted as indicators of the structural capacity for training and competence maintenance rather than as definitive classifications of individual proficiency.

### Robot-assisted repair

Robot-assisted groin hernia repair was identifiable in the database from the introduction of robotic platforms onward but accounted for only a minor proportion of procedures, predominantly TAPP-robot. Robotic procedures were classified within the minimally invasive category and did not materially affect the overall distribution between open and laparoscopic techniques.

### Long-term re-operation rates

**Fig. 5:**
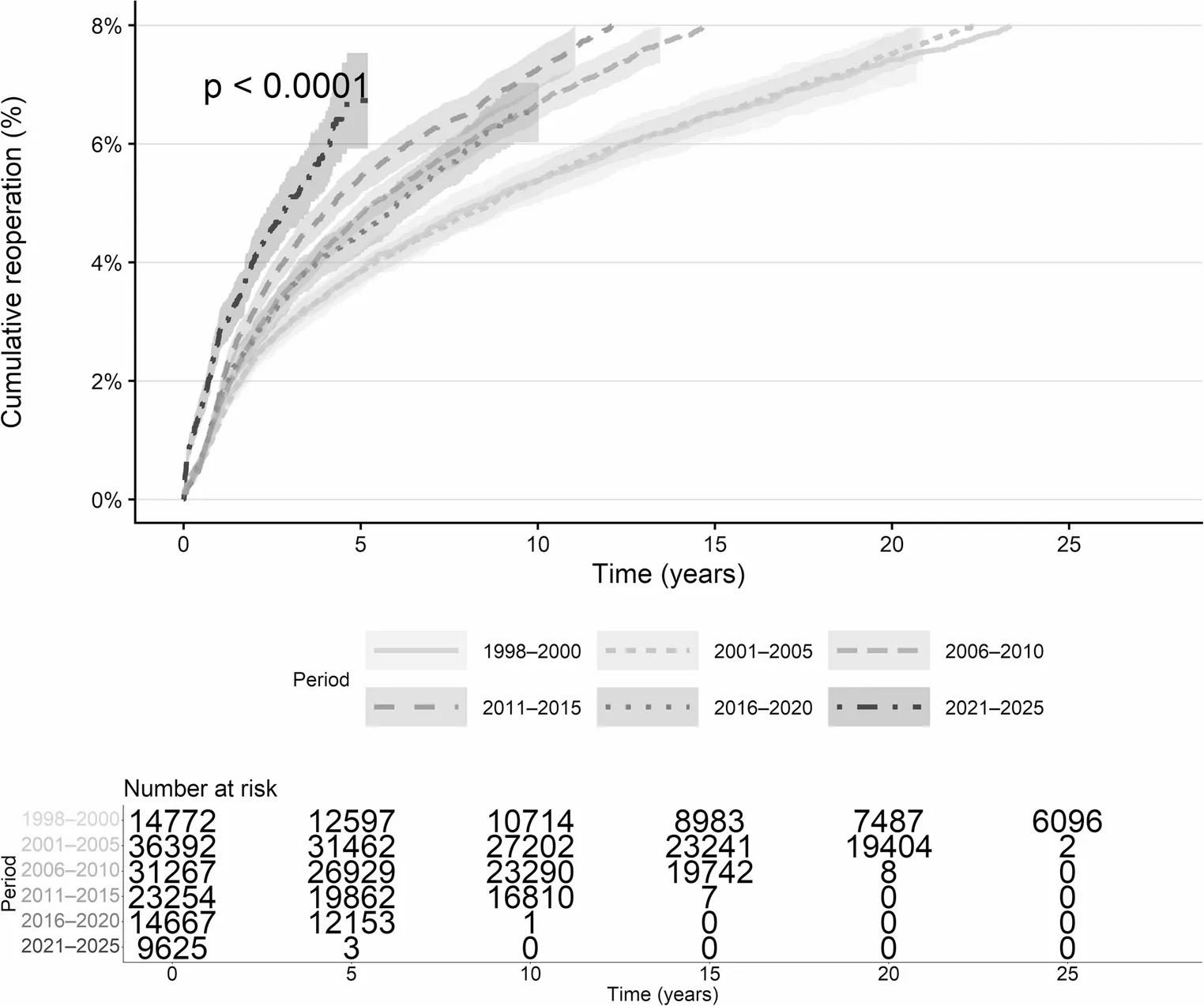
Kaplan–Meier plot showing cumulative re-operation rates after primary unilateral Lichtenstein repair, grouped by five-year period of primary surgery. Shaded areas indicate 95% confidence intervals; *P*-value derived from the log-rank test

**Fig. 6:**
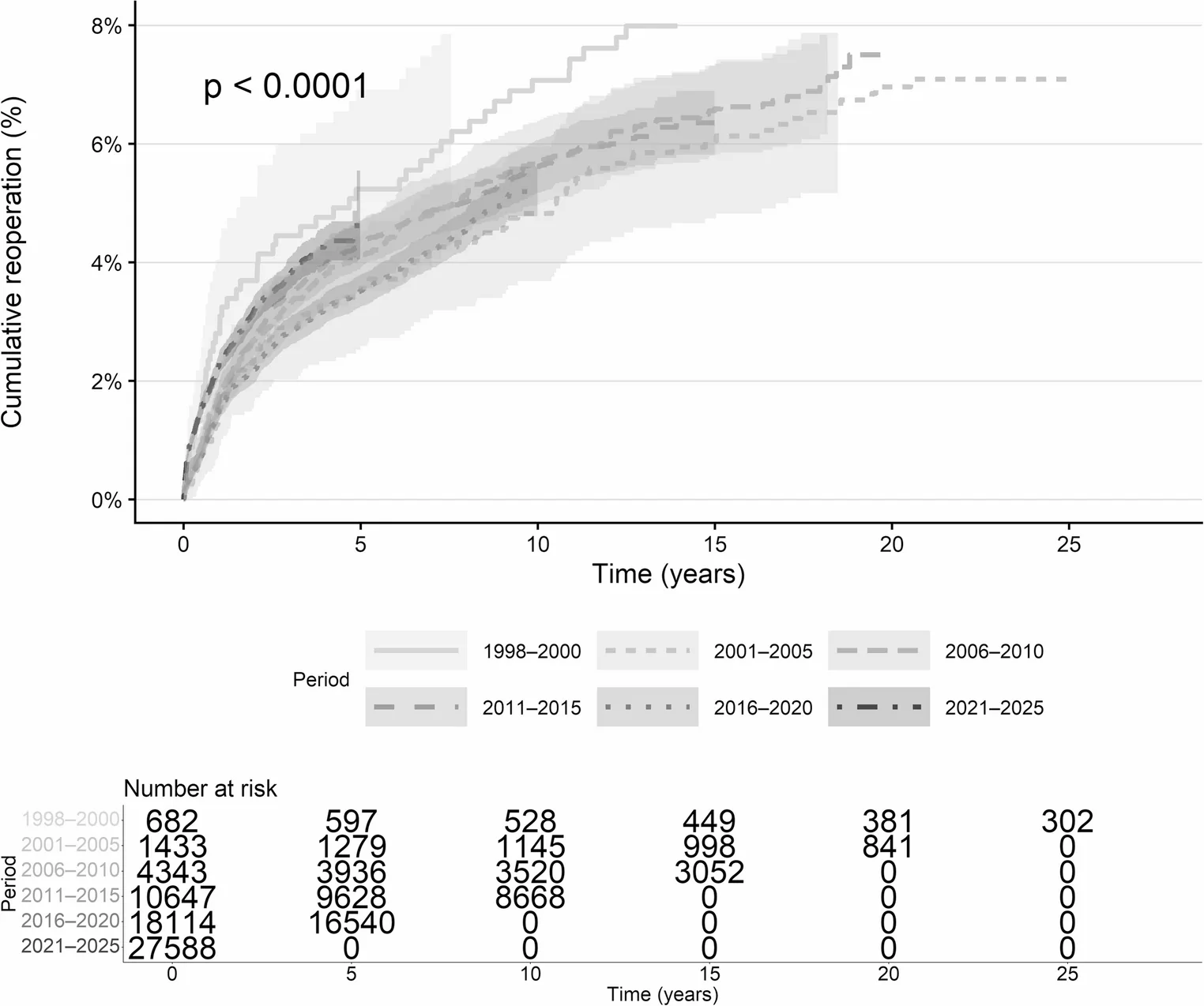
Kaplan–Meier plot showing cumulative re-operation rates after primary unilateral transabdominal preperitoneal (TAPP) repair, grouped by five-year period of primary surgery. Shaded areas indicate 95% confidence intervals; *P*-value derived from the log-rank test

Cumulative reoperation rates after primary unilateral Lichtenstein repair, stratified by five-year periods of primary surgery, are shown in Fig. 5. Reoperation for recurrence has increased significantly over time, with the most recent five-year cohort showing a higher cumulative reoperation rate than earlier cohorts (log-rank *P* < 0.0001). In contrast, reoperation rates after primary unilateral TAPP repair did not show the progressive increase observed after Lichtenstein repair across five-year strata (log-rank *P* < 0.0001; Fig. 6). Given the large sample size, even small absolute differences between strata may reach statistical significance; this finding should therefore be read as the absence of the marked, consistent deterioration seen after Lichtenstein repair rather than as perfect numerical stability.

To complement the Kaplan–Meier curves, crude two-year reoperation rates for primary unilateral Lichtenstein and TAPP repair are reported for the beginning, midpoint, and most recent five-year period of the registry. Lichtenstein 1998–2002: 2.35%; 2011–2015: 3.14%; 2021–2025: 3.45%; TAPP for the corresponding periods: 3.47%, 2.71%, 2.69%, each at two years of follow-up.

## Discussion

This nationwide analysis shows that open groin hernia repair has become a rare procedure in Denmark, both in relative and absolute terms. While the transition toward laparoscopic repair has not been accompanied by any apparent deterioration in overall outcomes, it has fundamentally altered the educational landscape of hernia surgery. Open repair, once a cornerstone of general surgical training, is now performed so infrequently and is so unevenly distributed across departments that maintaining broad competence through traditional, decentralized training pathways no longer seems realistic. It is also striking that reoperations for recurrence after Lichtenstein repair have increased significantly in the past five years compared with earlier periods.

The most important observation is not merely the declining proportion of open repairs but the very low nationwide absolute number of procedures, combined with their concentration in a small number of departments. Of the 40 Danish departments performing elective Lichtenstein repair in 2025, only four performed more than 100 repairs annually, while nearly half performed fewer than 20. Surgical competence is volume-dependent, and low procedural frequency poses well-recognized challenges for skill acquisition, retention, and quality assurance. When the remaining open repairs are dispersed across many small-volume centers, exposure becomes fragmented and inconsistent, limiting meaningful training opportunities for both trainees and early-career consultants.

What constitutes a “high” or “low” volume in open inguinal hernia repair is not formally defined, and no internationally agreed threshold for sustained procedural competence exists for this specific operation. Evidence from learning-curve and volume–outcome research nonetheless indicates that proficiency in open mesh repair, including reliable identification and handling of the inguinal nerves, continues to improve with cumulative experience and is unlikely to be acquired or maintained at very low annual caseloads [9, 17]. In the absence of a procedure-specific standard, we framed departmental volume descriptively against the observed national distribution rather than against an absolute cut-off. The concern is therefore not that a single numerical threshold has been crossed, but that a large proportion of departments now operate at the extreme low end of any plausible competence range – fewer than 20 cases per year – at which structured training and quality assurance become difficult regardless of where the precise threshold is set.

A particularly concerning finding is the parallel deterioration in long-term outcomes after Lichtenstein repair. Cumulative reoperation rates have increased significantly across consecutive five-year strata, whereas TAPP repair did not show a comparable deterioration. Although register-based reoperation rates underestimate true recurrence, they are robust and validated surrogate markers [6, 16], and a real upward trend is plausible in a setting where individual and departmental case volumes have fallen markedly. Other contributors – including evolving mesh choices [18], a shift in case mix toward more complex hernias, or changes in indications for open repair – cannot be excluded, but the temporal coincidence with declining absolute volume strongly supports a volume–outcome relationship.

The residual open groin hernia repairs no longer represent straightforward cases. They increasingly involve older patients with comorbidity, prior abdominal surgery, recurrent disease, or complex anatomy, including situations where laparoscopic repair is contraindicated or technically demanding. Such cases are inherently less suitable as educational operations, and their clinical management often extends beyond the operation itself, with non-operative considerations and patient-reported outcomes assuming greater importance [15, 19]. Patients who develop chronic post-herniorrhaphy pain – a recognized long-term complication of open mesh repair [20, 21] – also require longitudinal expertise that is unlikely to be sustained in low-volume settings.

These developments raise a fundamental question: who should perform open groin hernia repairs in the future? If current trends persist, most general surgeons will not encounter enough cases to maintain proficiency in open surgical cases [22]. Rather than a universally taught skill, open groin hernia repair appears to be transitioning into a specialist procedure performed by a limited number of dedicated surgeons. International experience with hernia registries and databases underscores that meaningful comparison and quality improvement depend on adequate surgeon and center volume [17, 23]. Decentralizing a small national volume across many departments risks eroding both quality and training value.

These findings should be interpreted in the context of the particular Danish setting, in which operative technique for elective groin hernia repair is unusually homogeneous. In Denmark, practice has effectively converged on two procedures – Lichtenstein and TAPP – and, most recently, predominantly on TAPP. Surgical practice in many other countries remains considerably more varied. In a registry-based, propensity-score-matched analysis of 109,130 primary unilateral inguinal hernia repairs from the Herniamed registry between 2019 and 2024, Lichtenstein (33,266), TEP (29,847), and TAPP (46,017) each accounted for a substantial share of procedures [24]. Similarly, in a French Hernia Club cohort of 21,976 patients operated between 2012 and 2019, open mesh repair still represented 43.6% of repairs alongside 54% laparoscopic procedures (and 2.4% without mesh) [25]. Where open repair retains a large and broadly distributed caseload, individual and departmental volumes may remain sufficient to support training and competence, and the case for centralisation is correspondingly less pressing. The educational and organisational imperative identified here may therefore be most acute in homogeneous, high-laparoscopic-uptake systems such as Denmark, and should not be assumed to apply uniformly to healthcare systems with a more diverse procedural mix.

From an educational perspective, the findings challenge long-standing assumptions about surgical training. The traditional model – in which open Lichtenstein repair serves as an introductory operation for trainees – is no longer aligned with contemporary practice. Laparoscopic groin hernia repair, performed in high volumes nationwide and with stable long-term outcomes, is better suited as a core component of basic surgical training. Structured training in TAPP and TEP, including simulation-based components, should be prioritized, and open repair should be reframed as an advanced technique reserved for selected surgeons in dedicated environments [2].

**Fig. 7:**
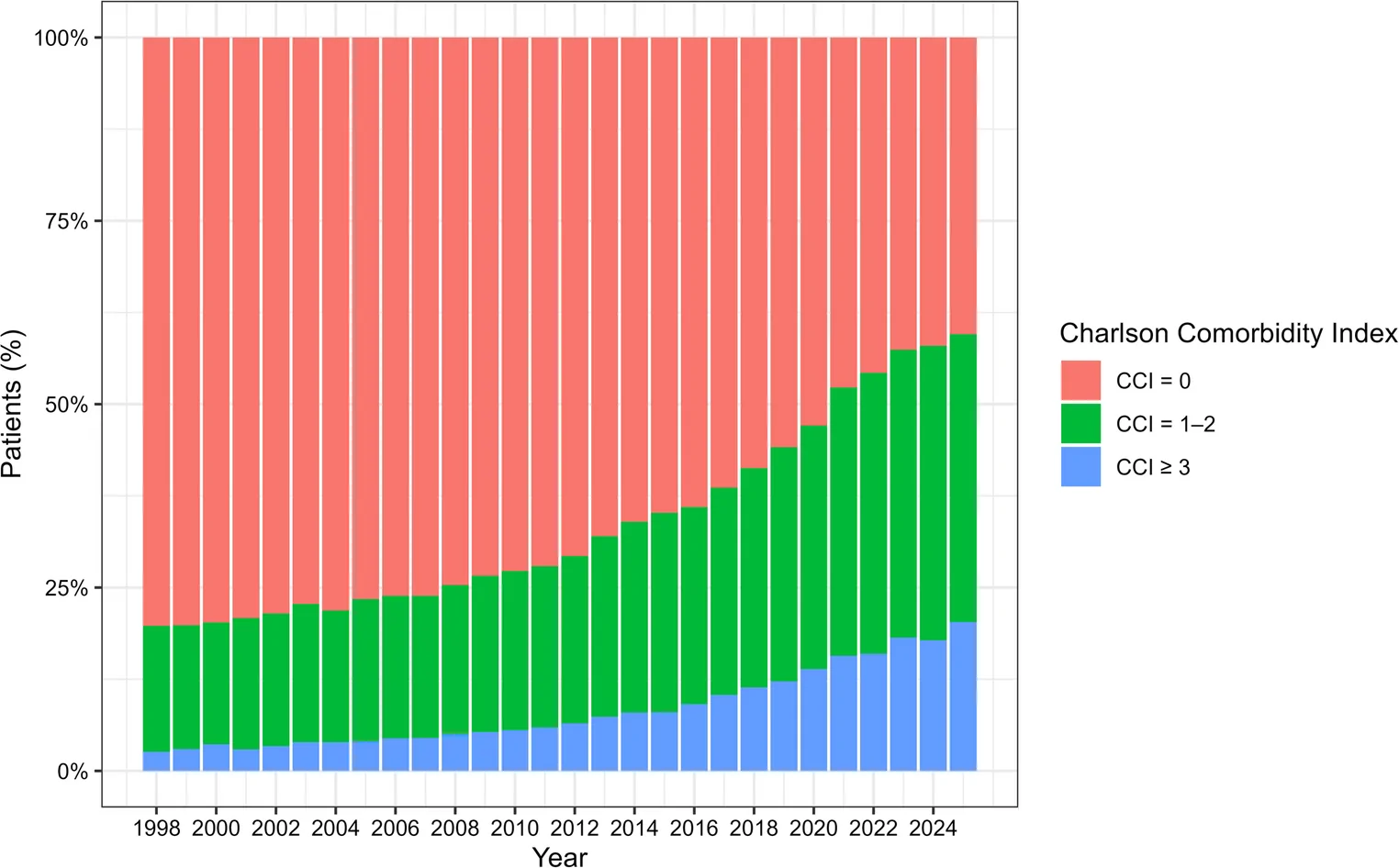
The change in case-mix comorbidity with time. The graphs shows that the Charlson comorbidity index increases over time in patients having Lichtenstein groin hernia repair

This study has limitations. Surgeon-level data on individual case volume were not available in the dataset analyzed, precluding precise estimation of individual learning curves and volume thresholds. Reoperation for recurrence is a conservative surrogate marker and may underestimate true recurrence rates, although it is well validated in Danish registry research [6, 16]. Registry data also do not capture informal training exposure, intra-departmental supervision, or simulation-based learning. Finally, our analysis is restricted to the Danish setting; the generalizability of the findings to healthcare systems with different organizational structures or referral patterns warrants confirmation. These limitations are nonetheless unlikely to alter the central observation that the national volume of open groin hernia repair is now too low to support broad-based training under the existing decentralized model. Two further limitations deserve emphasis. First, because the database does not record the number of surgeons operating within each department, departmental volume is an imperfect surrogate for individual surgeon exposure; some lower-volume departments may concentrate their open repairs among one or two dedicated surgeons who thereby maintain adequate personal caseloads, and center-level thresholds should be read with this caveat. Second, the rising reoperation rate after Lichtenstein repair may be influenced by selection bias and not by declining technique alone: as open repair has become concentrated in older, comorbid patients (see Fig. 7) with recurrent or anatomically complex hernias, an evolving case mix could itself contribute to the observed trend in recurrence. The dataset analysed did not permit a full comparison of comorbidity, hernia size, primary versus recurrent status, operative details, and operative time across time periods. We have therefore framed the volume–outcome association as plausible but not definitive and cannot exclude a contribution from changing patient selection.

Centralizing open groin hernia repair is a controversial but potentially necessary response to this reality. Concentrating the remaining open procedures in a limited number of centers or with designated surgeons could help preserve procedural quality, enable structured supervision, and create realistic training opportunities for those pursuing specialist hernia surgery. While centralization may seem counterintuitive for a historically common operation, it aligns with broader surgical trends in which low-volume, high-complexity procedures are increasingly regionalized to safeguard outcomes.

In conclusion, the near-universal adoption of laparoscopic repair has reshaped not only clinical practice but also the educational foundations of groin hernia surgery in Denmark. The simultaneous rise in reoperation rates after Lichtenstein repair raises legitimate concerns about quality in low-volume settings. Failure to address the resulting mismatch between training expectations and procedural reality risks undermining both surgical education and patient safety. A deliberate move toward centralizing open hernia surgery, combined with structured laparoscopic training as the new educational core, deserves serious consideration. This imperative is likely to be most pressing in countries with a homogeneous, laparoscopy-dominated practice such as Denmark; in healthcare systems where open repair remains common and broadly distributed, the optimal organisational and educational response may differ, and our conclusions should be applied with that caveat.

### Educational implications

Box 1. Educational implications of declining open groin hernia repair volumes in Denmark.

- Open groin hernia repair is no longer a high-volume routine procedure in Denmark.
- National absolute volume is insufficient to support broad-based training under current decentralized models.
- Remaining open repairs are increasingly complex and poorly suited as introductory teaching operations.
- Cumulative re-operation rates after Lichtenstein repair have risen, while TAPP outcomes did not show a comparable rise.
- Decentralized performance risks low surgeon-specific volumes and erosion of expertise.
- Centralization of open repair to dedicated centers or designated surgeons may be required.
- Laparoscopic repair should be prioritized as the new core procedure for surgical training.

## Data Availability

Aggregated data are presented in the manuscript. Individual-level data from the Danish Inguinal Hernia Database cannot be shared under Danish law.
